# Supplementary material for: Accelerating the energy transition towards photovoltaic and wind in China
Source: Nature. 2023 Jul 26;619(7971):761–7. doi: 10.1038/s41586-023-06180-8 (PMC10371865; doi:10.1038/s41586-023-06180-8)
Supplement: Supplementary file 1 — This file includes Supplementary Data Set 1, Supporting Methods 1–9, Supporting Figures 1–5 and Supporting Tables 1–10 [file 41586_2023_6180_MOESM1_ESM.pdf]

nature portfolio

https://doi.org/10.1038/s41586-023-06180-8

Supplementary information

Accelerating the energy transition towards photovoltaic and wind in China

In the format provided by the authors and unedited

Supplementary Materials for

Accelerating the energy transition towards photovoltaic and wind in China

Yijing Wang[1], Rong Wang[1,2,3,4,5,6*], Katsumasa Tanaka[7,8], Philippe Ciais[7,9], Josep Penuelas[10,11], Yves Balkanski[7], Jordi Sardans[10,11], Didier Hauglustaine[7], Wang Liu[1], Xiaofan Xing[1], Jiarong Li[1], Siqing Xu[1], Yuankang Xiong[1], Ruipu Yang[1], Junji Cao[12], Jianmin Chen[1,2,3], Lin Wang[1,2,3], Xu Tang[2,3], Renhe Zhang[2,3]

[1]Shanghai Key Laboratory of Atmospheric Particle Pollution and Prevention (LAP[3]), Department of Environmental Science and Engineering, Fudan University, Shanghai 200438, China.

[2]IRDR International Center of Excellence on Risk Interconnectivity and Governance on Weather/Climate Extremes Impact and Public Health, Fudan University, Shanghai 200438, China.

[3]Institute of Atmospheric Sciences, Fudan University, Shanghai 200438, China.

[4]Shanghai Frontiers Science Center of Atmosphere-Ocean Interaction, Shanghai, China.

[5]MOE Laboratory for National Development and Intelligent Governance, Fudan University, Shanghai, China.

[6]Institute of Eco-Chongming (IEC), Shanghai, China.

[7]Laboratoire des Sciences du Climat et de l'Environnement (LSCE), CEA/CNRS/UVSQ, Université Paris-Saclay, Gif-sur-Yvette 91190, France.

[8]Earth System Division, National Institute for Environmental Studies (NIES), Tsukuba 305-8506, Japan.

[9]Climate and Atmosphere Research Center (CARE-C) The Cyprus Institute 20 Konstantinou Kavafi Street, 2121, Nicosia, Cyprus.

[10]CSIC, Global Ecology Unit CREAF-CSIC-UAB, Bellaterra, Catalonia 08193, Spain.

[11]CREAF, Cerdanyola del Vallès, Catalonia 08193, Spain.

[12]Institute of Atmospheric Physics, Chinese Academy of Sciences, Beijing 100029, China.

[*]Correspondence: Rong Wang (rongwang@fudan.edu.cn).

Table of Contents

Supplementary Data Set 1

Supporting Methods 1–9

Supporting Figures 1–5

Supporting Tables 1–10

Supplementary data set

Supplementary Data Set 1. The data of ultra-high-voltage transmission lines. The data of ultra-high-voltage transmission lines constructed by 2060.

Supporting Methods

S1. Geospatial data used in the optimisation model

We compiled the data of land-cover at a spatial resolution of 0.005°×0.005° from the Moderate Resolution Imaging Spectroradiometer (MODIS) Land Cover Type 1 (MCD12Q1) data set (United States Geological Survey, 2014). All land pixels were categorized into forests, shrublands, savanna, grassland, wetland, croplands, urban and built-up lands, natural vegetation mosaics, snow and ice, desert and water bodies. The suitability of installation of PV panels or onshore wind turbines was defined based on land-cover in each pixel (**Supplementary Table 3**).

To estimate the area of pixels suitable for installing PV panels and onshore-wind turbines for power generation, we compiled the area of terrestrial ecological reserve at a spatial resolution of 0.001°×0.001° from the Resource and Environment Science and Data Center (Resource and Environment Science and Data Center, 2020), the slope of ground at a spatial resolution of 0.001°×0.001° from the Shuttle Radar Topography Mission (SRTM) global enhanced slope data set (United States Geological Survey, 2015), and the zero-plane displacement height and the surface roughness at a spatial resolution of 0.5°×0.625° from the NASA's Goddard Earth Observing System Model, version 5 (GEOS-5) Forward Processing (FP) database (Global Modeling and Assimilation Office, 2021).

To estimate the area of pixels suitable for installing offshore-wind turbines for power generation, we compiled the area of China's territorial sea from the Maritime Boundaries Geodatabase (Flanders Marine Institute, 2018), depth of water from the Radar Topography Mission (SRTM) Global Enhanced Slope Database (United States Geological Survey, 2015), routes of shipping from the Modern-Era Retrospective analysis for Research and Applications, version 2 (MERRA-2) database (Gelaro et al., 2017), and the area of marine ecological reserve from the National Marine Data and Information Service (UN Environment Programme World Conservation Monitoring Centre, 2021; Resource and Environment Science and Data Center, 2020).

We compiled the hourly solar radiation and surface air temperature during 2012–2018 at a spatial resolution of 0.25° in latitude and 0.31° in longitude from the NASA's Goddard Earth Observing System Model, version 5 (GEOS-5) Forward Processing (FP) database (Global Modeling and Assimilation Office, 2021), which were re-projected on the map at a spatial resolution of 0. 0083° in latitude and 0.033° in longitude by assuming the homogeneity within the pixel of 0.25°×0.31°. We compiled the hourly ground friction velocity of wind speed at a spatial resolution of 0.5° in latitude and 0.625° in longitude during 2012–2018 from the MERRA-2 data set (Gelaro et al., 2017), which were re-projected on the map at a spatial resolution of 0. 0083° in latitude and 0.033° in longitude by assuming homogeneity within the pixel of 0.5°×0.625°.

We compiled data of energy consumption in the power, residential, industry, transportation and other sectors by province in 2019 from the Chinese Energy Statistical Yearbook 2020 (National Bureau of Statistics of China, 2020). The amounts of energy consumption are projected by sector and by province from 2021 to 2060 based on the energy consumption in 2020 and the rate of growth of energy demand in China by year according to the projection by the International Energy Agency (International Energy Agency, 2021). We disaggregated the energy consumptions from 31 provinces over the mainland to 2,373 counties using the spatial distribution of GDP at a spatial resolution of 0.01°×0.01° in 2019 from the Resource and Environment Science and Data Center (REDC) (Xu, 2017) due to a lack of data for Hongkong, Macao and Taiwan.

Geospatial data used in the optimisation model are summarized in **Supplementary Table 2**.

S2. Calculation of wind speed at a hub height

Wind power is subject to large seasonal and diurnal variabilities. To deliver a representative estimate of wind power resources for 2021–2060, we estimated the hourly wind speed at a hub height of 100 meters above ground in the period for 2012–2018 using a log-law function (Rinne et al., 2018):

$$V=\frac{u^*}{k}\ln\left(\frac{h-d}{z_0}\right) \tag{1}$$

where V is wind speed at a height (h) of 100 meters above ground and k is the Von Karman constant (0.41). The hourly friction velocity of wind speed (u^*) was compiled from the MERRA-2 data set at a spatial resolution of 0.5°×0.625° (Gelaro et al., 2017), while the zero-plane displacement height (d) and the surface roughness (z_0) were compiled from the NASA's GEOS-5 FP database at a spatial resolution of 0.5°×0.625° (Global Modeling and Assimilation Office, 2021).

S3. Power generation by photovoltaic-power plants

We identified the area of pixels suitable for utility-scale power generation by photovoltaic (PV) plants with a capacity of >10 MW using solar energy based on the type of land-cover, ground slope, solar radiation, surface air temeperature and the proven area of terrestrial ecological reserve. We defined the suitability factor for suitable lands varying from 10 to 15% based on the land-cover, while we excluded the area of natural reserve with ecological functions and the pixels with ground slope >5%, solar radiation <4.2 hour per d and surface atmospheric temperature <0°C (**Supplementary Table 3**).

Electricity generation by PV-power plants is influenced by solar radiation, the effective area of PV panels, the geographic location of PV panel, surface atmospheric temperature, the shade between PV panels and the efficiency of energy conversion (Chen et al., 2021). We estimated the capacity potential of PV panel (P_{PV}) (Masters, 2013):

$$P_{PV}=S_{panel}\times\Omega\times SR \qquad (2)$$

where S_{panel} is the area of pixels installed with PV panels, Ω is the ratio of effective panel area to the area of pixels installing with PV panels, and SR is the standard power capacity of PV panels (161.9 Watt per m^2) (Masters, 2013).

We estimated the hourly power generation (W_{PV}) by a PV-power plant as a function of capacity potential (P_{PV}) (Masters, 2013; Chen et al., 2019):

$$W_{PV}=P_{PV}\times\frac{I_{panel}}{I_0}\times\gamma_{shade}\times\gamma_{temp}\times\left(1-\gamma_{loss}\right) \qquad (3)$$

where I_{panel} is solar radiation captured by PV panels, I_0 is intercepted radiation of PV panels (1,000 Watt per m^2) under a standard test condition, γ_{shade} is the shade coefficient, γ_{temp} is the

temperature coefficient, and γ_{loss} is electricity loss from power generation to grid connection (19.44%). We estimated I_{panel}, γ_{shade} and γ_{temp} using the recommended methods (Masters, 2013; Chen et al., 2019), which are described below.

First, we estimated solar radiation (I_{panel}) captured by PV panels as the sum of direct (I_{direct}), diffuse (I_{diff}) and reflected radiation (I_{ref}) (Masters, 2013):

$$I_{panel}=I_{direct}+I_{diff}+I_{ref}=(\cot\beta\cos\phi\sin\omega+\cos\omega)R_{direct}+\frac{1+\cos\omega}{2}R_{diff}+\frac{1-\cos\omega}{2}\rho_s R_{total} \quad (4)$$

where ρ_s is the surface albedo (0.2) (Chen et al., 2019). The hourly solar direct (R_{direct}), diffuse (R_{diff}) and total (R_{total}) radiation were compiled from the NASA's GEOS-5 FP database at a spatial resolution of 0.25°×0.31° (Global Modeling and Assimilation Office, 2021). It should be noted that there is an ongoing debate on how the surface solar radiation has changed in different regions (Wild et al., 2015) and what has caused the regional change in surface solar radiation (i.e. effects of aerosols or cloud cover) (Imamovic et al., 2016). However, it is also known that historical changes in surface solar radiation are not well reproduced by climate models, thus limiting our ability for predicting future changes in surface solar radiation (Moseid et al., 2020).

By assuming that the fixed-tilt PV panels are used to generate electricity, we estimated the ratio of the effective panel area to the area of pixels installed with PV panels (Ω) (Masters, 2013):

$$\Omega=\left(L_p\times\cos\omega+L_p\times\frac{\sin\omega}{\tan\beta}\times\cos\phi\right)^{-1} \quad (5)$$

where the tilt degree of PV panel (ω), the solar altitude angle (β) and the solar azimuth (ϕ) were calculated as:

$$\omega=\frac{\pi}{180}\left(a_0+a_1\theta+a_2\theta^2+a_3\theta^3\right) \quad (6)$$

$$\beta=\mathrm{asin}(\sin\theta\times\sin\delta+\cos\theta\times\cos\delta\times\cos\varphi) \quad (7)$$

$$\phi=acos\frac{\sin\beta\times\sin\theta-\sin\delta}{\cos\beta\times\cos\theta} \quad (8)$$

$$\delta=b_0+b_1\cos d_A+c_1\sin d_A+b_2\cos 2d_A+c_2\sin 2d_A+b_3\cos 3d_A+c_3\sin 3d_A \quad (9)$$

$$d_A=\frac{2\pi(t_d\text{-}1)}{365} \quad (10)$$

$$\varphi=15\times(t_{hour}+\frac{t_{min}}{60}+\frac{4\times(\theta\text{-}120)}{60}-12) \quad (11)$$

where θ is the latitude of the installed solar panel, δ is the solar declination angle, φ is the solar hour angel, t_d is the day (1 to 365), t_{hour} is the hour of time (1 to 24) and t_{min} is the minute of time (1 to 60). The coefficients a_0 (1.3793), a_1 (1.2011), a_2 (–0.0144) and a_3 (8.051×10^{-5}) were estimated in a previous study (Jacobson and Jadhav, 2018), while the coefficients b_0 (0.00692), b_1 (–0.400), c_1 (0.0703), b_2 (–0.006768), c_2 (0.000907), b_3 (–0.00270) and c_3 (0.00148) were estimated in a previous study (Masters, 2013).

Second, we estimated the shade coefficient (γ_{shade}) to consider the impact of shading between PV panels on the efficiency of solar energy capture (Chen et al., 2019):

$$\gamma_{shade} = 11/12 - \left(\sin\omega\cot\beta\cos\phi - \frac{\sin\omega}{\tan\beta}\cos\phi\right)\frac{\sin\left(\mathrm{atan}\frac{\tan\beta}{\cos\phi}\right)}{\sin\left(\pi-\mathrm{atan}\frac{\tan\beta}{\cos\phi}-\omega\right)} \quad (12)$$

where γ_{shade} is the proportion of the unshaded area relative to the total area of PV panels.

Third, we estimated the temperature coefficient (γ_{temp}) be considering the negative effect of extremely high temperatures on the efficiency of converting solar energy into electricity (Kaldellis et al., 2014; Kawajiri et al., 2011):

$$\gamma_{temp} = 1 + \sigma_T\left[T_{atm} + \left(\frac{T_{cell}-20}{0.8}\right)I_{panel} - 25\right] \quad (13)$$

wher T_{atm} is the hourly atmospheric surface temperature at 2 meters above ground, T_{cell} is the normal cell operating temperature (44 °C) and σ_T is the temperature coefficient (–0.41% per °C) (Chen et al., 2019).

S4. Power generation by onshore wind-power plants

We identified the area of pixels suitable for utility-scale power generation by onshore-wind power plants with a capacity of >10 MW based on the type of land-cover, ground slope, altitude of sites above sea-level and area of terrestrial ecological reserve (**Supplementary Table 3**). In our assumption, onshore wind-power plants are installed with the General Electric wind turbine at a maximal capacity of 2.5 MW at a hub height of 100 meters above ground to convert air kinetic energy to electricity. The power generation curve as a function of wind speed at a height of 100 meters for the General Electric model in the Wind-turbines database (https://en.wind-turbine-models.com/turbines) was adopted to estimate the capacity factor of onshore wind-power generation (Lu et al., 2020; Bauer and Matysik, 2021) (**Supplementary Fig. 3**).

Following a method in the literature (Lu et al., 2020), we estimated the onshore wind-power density ($\rho_{onshore}$) by installing wind turbines with an 8×8 rotor diameter to improve the efficiency of kinetic energy conversion with a low turbine-turbine interference on lands:

$$\rho_{onshore} = \frac{PW_{onshore}}{8D_{onshore} \times 8D_{onshore}} \quad (14)$$

where $PW_{onshore}$ is the maximal power of onshore-wind turbine (2.5 MW), and $D_{onshore}$ is the diameter of rotor for onshore-wind turbines (103 meters).

Based on the onshore-wind power density ($\rho_{onshore}$) and the area of pixels installed with onshore wind turbines ($S_{onshore}$), we estimated the capacity potential of onshore-wind power ($P_{onshore}$) (Masters, 2013):

$$P_{onshore} = S_{onshore} \times \rho_{onshore} \quad (15)$$

We estimated the hourly power generation by an onshore wind-power plant ($W_{onshore}$):

$$W_{onshore} = P_{onshore} \times CF_{onshore} \times U_{TI} \times A_{RR} \quad (16)$$

where $CF_{onshore}$ is the capacity factor of onshore-wind turbines calculated by the power generation function, U_{TI} is the efficiency of energy conversion (0.95) (Rinne et al., 2018), and A_{RR} is the array efficiency factor (0.9) (Rinne et al., 2018).

S5. Power generation by offshore wind-power plants

We identified the pixels suitable for utility-scale power generation by offshore-wind plants with a capacity of >10 MW using kinetic energy over the sea (Lu et al., 2020; Sherman et al., 2017). We considered that offshore-wind plants will be constructed in the oceans with water depth <60 meters (Becker et al., 2009) within China's Exclusive Economic Zone (Flanders Marine Institute, 2018), while the oceans with the frequent shipping routes (Gelaro et al., 2017) and the marine natural reserve (UN Environment Programme World Conservation Monitoring Centre, 2021; Resource and Environment Science and Data Center, 2020) were excluded for installing offshore-wind turbines. In our optimisation model, offshore wind-power plants are equipped with the Vestas wind turbine installed at a maximal capacity of 8 MW with a hub at a height of 100 meters above ground to generate electricity. The power curve as a function of wind speed at 100 meters above ground estimated by the Vestas model in the Wind-turbines database

(https://en.wind-turbine-models.com/turbines) was adopted to estimate the capacity factor of offshore wind power generation by Vestas (Lu et al., 2020; Bauer and Matysik, 2021) (**Supplementary Fig. 3**).

We estimated the power density ($\rho_{offshore}$) of offshore-wind turbines using a 7×7 rotor diameter to improve the efficiency of wind energy utilization with a low turbine-turbine interference on oceans (Lu et al., 2020; Sherman et al., 2017):

$$\rho_{offshore}=\frac{PW_{offshore}}{7D_{offshore}\times 7D_{offshore}} \quad (17)$$

where $PW_{offshore}$ is the maximal power of offshore wind turbine (8 MW), and $D_{offshore}$ is the diameter of rotor for offshore wind turbines (164 meters).

Based on the offshore-wind power density ($\rho_{offshore}$) and the area of pixels installing with offshore-wind turbines ($S_{offshore}$), we estimated the capacity potential of offshore-wind power ($P_{offshore}$) (Masters, 2013):

$$P_{offshore}=S_{offshore}\times\rho_{offshore} \quad (18)$$

We estimated the hourly power generation by an offshore wind-power plant ($W_{offshore}$):

$$W_{offshore}=P_{offshore}\times CF_{offshore}\times U_{TI}\times A_{RR}\times(1\text{-}F_{EN})\times(1\text{-}F_{LS}) \quad (19)$$

where $CF_{offshore}$ is the capacity factor of offshore-wind turbines calculated by the power generation function, U_{TI} is the efficiency of energy conversion (0.95) (Rinne et al., 2018) and A_{RR} is the array efficiency factor (0.9) (Rinne et al., 2018), F_{EN} is the fraction of electricity loss due to environmental and curtailment effects (0.02) (Musial et al., 2016), and F_{LS} is the fraction of electricity loss in the transmission from offshore wind turbines to onshore stations. We estimated the fraction of electricity loss in the transmission from offshore wind turbines to onshore stations (F_{LS}) using an empirical function (Musial et al., 2016):

$$F_{LS}=d_0+d_1D_L+d_2D_L^2+d_3D_L^3+d_4D_L^4+d_5D_L^5+e_1D_P+e_2D_P^2+e_3D_P^3+e_4D_P^4 \quad (20)$$

where D_L is the distance of an offshore wind turbine to an onshore station and D_P is the water depth. The coefficients d_0 (0.0207), d_1 (0.00073), d_2 (-1.6×10^{-5}), d_3 (1.7×10^{-7}), d_4 (-8.6×10^{-10}), d_5 (-1.57×10^{-12}), e_1 (0.000015), e_2 (-4.7×10^{-8}), e_3 (8.2×10^{-11}) and e_4 (-4.1×10^{-14}) were derived from a previous study (Musial et al., 2016).

S6. Costs of ultra-high-voltage transmission of electricity

We considered that electricity will be transported among regions in a national grid network of ultra-high-voltage (UHV) transmission using 130 lines that have been projected in China's national grid development plans and 817 lines that have not been projected but are needed in our optimisation model before 2060 (Center for Security and Emerging Technology, 2021) (see the detailed information on these UHV lines in the **Supplementary Spreadsheet S1**). The direct current (DC) UHV is designed at a capacity of 8,000 MW for ±800 kV DC and 12,000 MW for ±1,100kV DC, respectively (Chen et al., 2021). In contrast, the UHV transmission capacity of alternating current (AC) is a function of the transmission distance estimated in a previous study (Chen et al., 2021), where the capacity of 1,000 kV AC decreases from 6,000 MW for 100 km to 3,000 MW for 3,000 km. Based on the capacity and distance of electricity transmission, we estimated the costs of transmission (A_ϵ) (Chen et al., 2021):

$$A_\epsilon = \sum_{l=1}^{77} \left[\mu_{line} \cdot D_l \cdot \mathrm{int}\left(\frac{\max \Theta_{hl}}{P_{UHV}} \right) + \mu_{sub} \cdot \max \Theta_{hl} \right] \quad (21)$$

where l is a transmission line, μ_{line} is the line cost per kilometer ($732,220, $800,383 and $670,785 per km for ±800 kV DC, ±1,100kV DC and 1,000 kV AC, respectively) (Electric Power Planning and Engineering Institute, 2011; Electric Power Planning and Engineering Institute, 2020), D_l is the length of a transmission line, Θ_{hl} is the hourly electricity carried by a transmission line, P_{UHV} is the capacity of a transmission line (8,000 MW or 12,000 MW for DC and 3,000 to 6,000 MW for AC), and μ_{sub} is unit costs of converters for DC lines or substations for AC lines ($82, $92 and $41 per kW for ±800 kV DC, ±1,100kV DC and 1,000 kV AC, respectively) (Electric Power Planning and Engineering Institute, 2020).

S7. Costs of energy storage by hydro pump or chemical batteries

By considering that chemical batteries can be charged and discharged for 6,000 times over a lifetime of 15 years (Chen et al., 2021) and that pumped-hydro storage can be charged and discharged for 1 time per day over a lifetime of 50 years (Cole and Frazier, 2019), we estimated the costs of energy storage (G_ϵ):

$$G_\epsilon = \mu_{power} \cdot \sum_{q=1}^{7} \sum_{x=1}^{n_q} P_x + \mu_{throu} \cdot \frac{\sum_{h=1}^{8760} \Lambda_h}{N_c} + \mu_{opera} \cdot \sum_{h=1}^{8760} \Lambda_h \cdot (1+\varepsilon) \cdot \sum_{t=1}^{t} \frac{1}{(1+r)^t} \quad (22)$$

where x is a power plant, h is an hour, q is a region, n_q is the number of power plants, P_x is the capacity potential of storage systems (maximal hourly electricity in storage), Λ_h is the hourly electricity in storage, μ_{power} is unit costs of power capacity ($1,200 per kW for pumped-hydro storage in 2020–2060; $595, $374, $327, $280 and $234 per kW for chemical battery storage in 2020, 2030, 2040, 2050 and 2060, respectively) (Hiesl et al., 2020; Cole and Frazier, 2019), μ_{throu} is unit costs of throughput in the storage systems ($100 per kWh for pumped-hydro storage in 2020–2060; $345, $198, $174, $149 and $124 per kWh for lithium battery storage in 2020, 2030, 2040, 2050 and 2060, respectively) (Chen et al., 2021; Cole and Frazier, 2019), μ_{opera} is unit operational costs in charging and discharging ($0.0015 per kWh) (Zhang et al., 2016), N_c is the number of charging and discharging (365 and 400 for pumped-hydro storage and chemical battery storage, respectively) (Chen et al., 2021), ε is the ratio of energy after charging and discharging (85%) (Cole and Frazier, 2019), t is the lifetime of storage (50 and 15 years for pumped-hydro storage and chemical batteries, respectively) (Chen et al., 2021; Cole and Frazier, 2019), and r is the discounting rate (5% per y) (Duan et al., 2021). By comparing LCOE for each power plant using mechanical (pumped hydro) or chemical (chemical batteries) storage, we sought the optimal option of energy storage to achieve the lower LCOE for each power plant.

S8. Intertemporal dynamics of learning

We adopted the formulation of learning by doing (Mcdonald and Schrattenholzer, 2001) to estimate the ratio of the declined capital costs (ξ_x) by accumulating low-carbon investments:

$$\xi_x=1-\left(\frac{\sum_{q=1}^{7}\sum_{\varsigma=1}^{n_q}P_\varsigma+P_0}{P_0}\right)^{\log_2(1-r_{LR})},\ t_\varsigma<t_x \quad (23)$$

where x is a new PV or wind-power plant, ς is a PV or wind-power plant build before x, t_x is the time of building plant x, t_ς is the time of building plant ς, P_ς is the power capacity of plant ς, q is a region, n_q is the number of power plants in a region, P_0 is the total capacity of PV or wind power built by 2020 (253,000 MW, 272,010 MW and 8,990 MW for PV, onshore wind and offshore wind, respectively) (China Energy Storage Network, 2021), and r_{LR} is the rate of learning. We derived the average of rates of learning (32.4% for PV panels and 11.78% for wind turbines, 18% for inverters, mounting materials, secondary equipment, installation work and

administration and grid connection, and 18% for power transmission) from measurements in China and considered their uncertainty ranges in our Monte Carlo simulations (**Supplementary Table 1**).

S9. Estimation of the Gini coefficient for income inequality

We estimated the Gini coefficient to represent income inequality (Yitzhaki, 1979) by dividing all population into 2,002 groups in the order of per capita income. We estimated the income Gini coefficient in China following a simple formula (Mi et al., 2020):

$$Gini = 1 - \sum_{i=1}^{n_i}\left[\left(2\sum_{g=1}^{i}\Gamma_g + \Gamma_i\right)\cdot\Psi_i\right] \quad (24)$$

where i is a population group in the country, n_i is the number of groups in the country (2,002), g is a group with per capita income lower than that in group i, Γ_g or Γ_i is the ratio of income in group g or i to the total income of the population in the country, and Ψ_i is the ratio of population in group i to the total population in the country. We estimated Γ_g, Γ_i and Ψ_i in the country based on the income distribution in 2,373 counties in China in 2060 by varying the price of carbon (ς) from \$0 to \$100 per tCO_2.

We estimated Γ_g, Γ_i and Ψ_i by considering the impact of finances embodied in the flow of PV and wind power based on income distribution of population in 2060. First, we estimated the income distribution of population by county based on the residents' income distribution for urban and rural population according to a national socioeconomic survey in 2015 (Zhang et al., 2016). According to this income distribution, we estimated the average per capita income of urban and rural population by county and thus the frequency distribution of per capita income among urban and rural populations. To estimate the income distribution of population by county, we divided the urban or rural population into 1,001 groups. This method generates a total of 2,002 groups for urban and rural populations in each county. Second, we compiled the per capita disposable income among urban and rural population in 2,373 counties in China in 2015–2019 from the Provincial Statistical Yearbook (National Bureau of Statistics of China, 2020), and predicted per capita disposable income in 2060 based on the projected growth rate of income by province in China during 2020–2060 (National Bureau of Statistics of China, 2020). We assumed that the growth rate of per capita income is the same for all groups in a county due to

the lack of data. We calibrated the growth rate of per capita income in each income group by county during 2015–2060 to ensure that the predicted per capita income as an average for each county in 2060 is equal to the projection in 2060 by the Provincial Statistical Yearbook (National Bureau of Statistics of China, 2020). This calculation returns the distribution of per capita income among rural and urban population by county in 2060.

Under a prescribed carbon price (ς), we sought for the power plants with the marginal abatement costs lower than this carbon price, returning the total revenue (R_ϵ) of power generation after building a new PV or wind-power plant (ϵ):

$$R_\epsilon = \varrho \cdot E_\epsilon + \zeta \cdot F_\epsilon - LCOE_\epsilon \cdot E_\epsilon \quad (25)$$

where ϱ is the price of coal, oil or gas substituted by PV or wind power, F_ϵ, E_ϵ and $LCOE_\epsilon$ are abated CO_2 emissions, power generation and LCOE after building plant ϵ. We then obtained the revenue in each county by building PV and wind-power plants as:

$$T_x = \sum_{\epsilon=1}^{n_x} (R_\epsilon - R_{\epsilon\text{-}1}), \quad \epsilon \in x \quad (26)$$

where x is a county building the power plant ϵ, and n_x is the number of PV and wind-power plants in this county.

We filtered pixels in urban area when building new PV or wind-power plants, so we allocated the revenue among the rural population in China as a part of poverty alleviation project in the country. By considering payments due to the increase in the costs of power generation after levying a carbon tax on fossil fuels, we estimated the per capita income of each population group in rural and urban areas:

$$INR_{i,x} = INR_{i,x,0} + \frac{T_x}{RUR_{i,x}} - \zeta \cdot \theta_{fossil} \cdot m_x \cdot u_{i,x} \quad (27)$$

$$INU_{i,x} = INU_{i,x,0} - \zeta \cdot \theta_{fossil} \cdot m_x \cdot v_{i,x} \quad (28)$$

where x is a county (x=1 to 2,373), i is a group in the rural or urban population (i=1 to 1,001), $INR_{i,x}$ is the per capita income for a rural group, $INU_{i,x}$ is the per capita income for a urban group, $INR_{i,x,0}$ is the per capita income for rural group x without building PV and wind-power plants, $INU_{i,x,0}$ is the per capita income for a urban group without building PV and wind-power plants, T_x is the revenue of building new PV and wind-power plants in this county, RUR_x is rural

population in county x, θ_{fossil} is CO_2-emission factor of fossil fuels (0.84 kg CO_2 per kWh for coal, 0.72 kg CO_2 per kWh for oil and 0.46 kg CO_2 per kWh for gas, respectively) (Liu et al., 2015a), m_x is per capita energy consumption in county x, ς is the carbon price, and $u_{i,x}$ or $v_{i,x}$ is a factor to change the ratio of per capita energy consumption in a rural or urban group relative to the per capita energy consumption in this county. By assuming that the per capita income was proportional to per capita energy consumption in each county (Mi et al., 2020), we derived the per capita energy consumption as a function of per capita income:

$$u_{i,x}=INR_{i,x,0}\cdot\frac{\sum_{i=1}^{1001}p_{i,x}+\sum_{i=1}^{1001}q_{i,x}}{\sum_{i=1}^{1001}\left(INR_{i,x,0}\cdot p_{i,x}\right)+\sum_{i=1}^{1001}\left(INU_{i,x,0}\cdot q_{i,x}\right)} \quad (29)$$

$$v_{i,x}=INU_{i,x,0}\cdot\frac{\sum_{i=1}^{1001}p_{i,x}+\sum_{i=1}^{1001}q_{i,x}}{\sum_{i=1}^{1001}\left(INR_{i,x,0}\cdot p_{i,x}\right)+\sum_{i=1}^{1001}\left(INU_{i,x,0}\cdot q_{i,x}\right)} \quad (30)$$

where $p_{i,x}$ is the number of population in a rural group, $q_{i,x}$ is the number of population in an urban group, $INR_{i,x,0}$ is the per capita income for rural group x without building PV and wind-power plants, and $INU_{i,x,0}$ is the per capita income for a urban group without building PV and wind-power plants. The parameters $p_{i,x}$ and $q_{i,x}$ were derived from the projected income distribution by county in 2060. The parameters $INR_{i,x,0}$ and $INU_{i,x,0}$ were determined based on the division of population based on the per capita income.

Supporting Figures

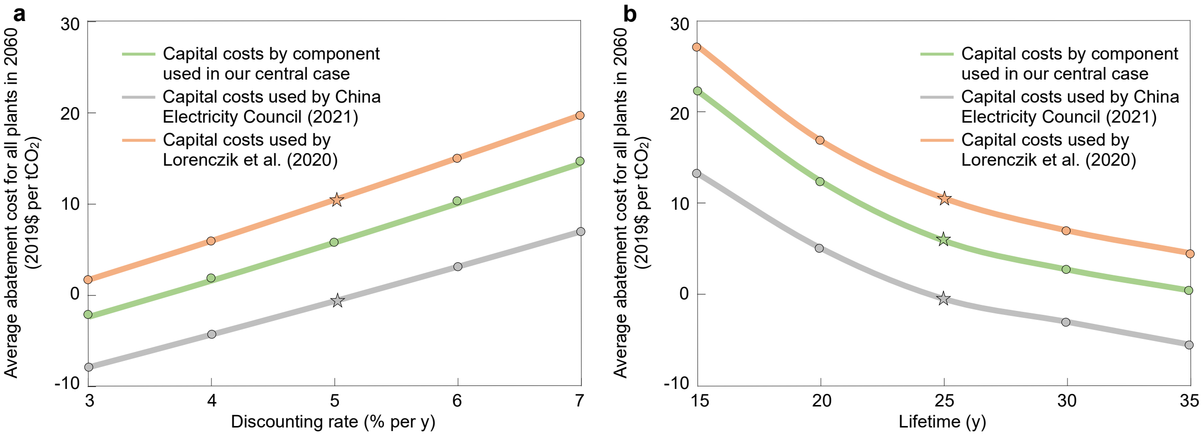

Fig. S1. Impacts of the discounting rate and lifetime of power plants on the average marginal abatement cost (MAC) for building all new PV and wind power-plants during 2021–2060 under different capital costs. We estimate the average MAC when increasing the discounting rate from 3 to 7% per y (**a**) or increasing the lifetime of power plants from 15 to 35 years (**b**) under different capital costs (Lorenczik et al., 2020; China Electricity Council, 2021; China Photovoltaic Industry Alliance, 2021; Liu et al., 2015b). The capital costs ($0.73 and $0.88 per Watt for PV and onshore wind, respectively) published by Lorenczik et al. (2020) (orange line) are higher than the data ($0.23 and $0.76 per Watt for PV and onshore wind, respectively) published by the China Electricity Council (2021) (gray line). In our central case (green line), we adopt the capital costs by component using the data published by the China Photovoltaic Industry Alliance (2021) for PV-power plants ($0.64 per Watt as the total capital costs) and using the data by Liu et al. (2015b) for onshore wind-power plants ($0.68 per Watt as the total capital costs), because neither of the two estimates above (Lorenczik et al., 2020; China Electricity Council, 2021) provides the capital costs by component.

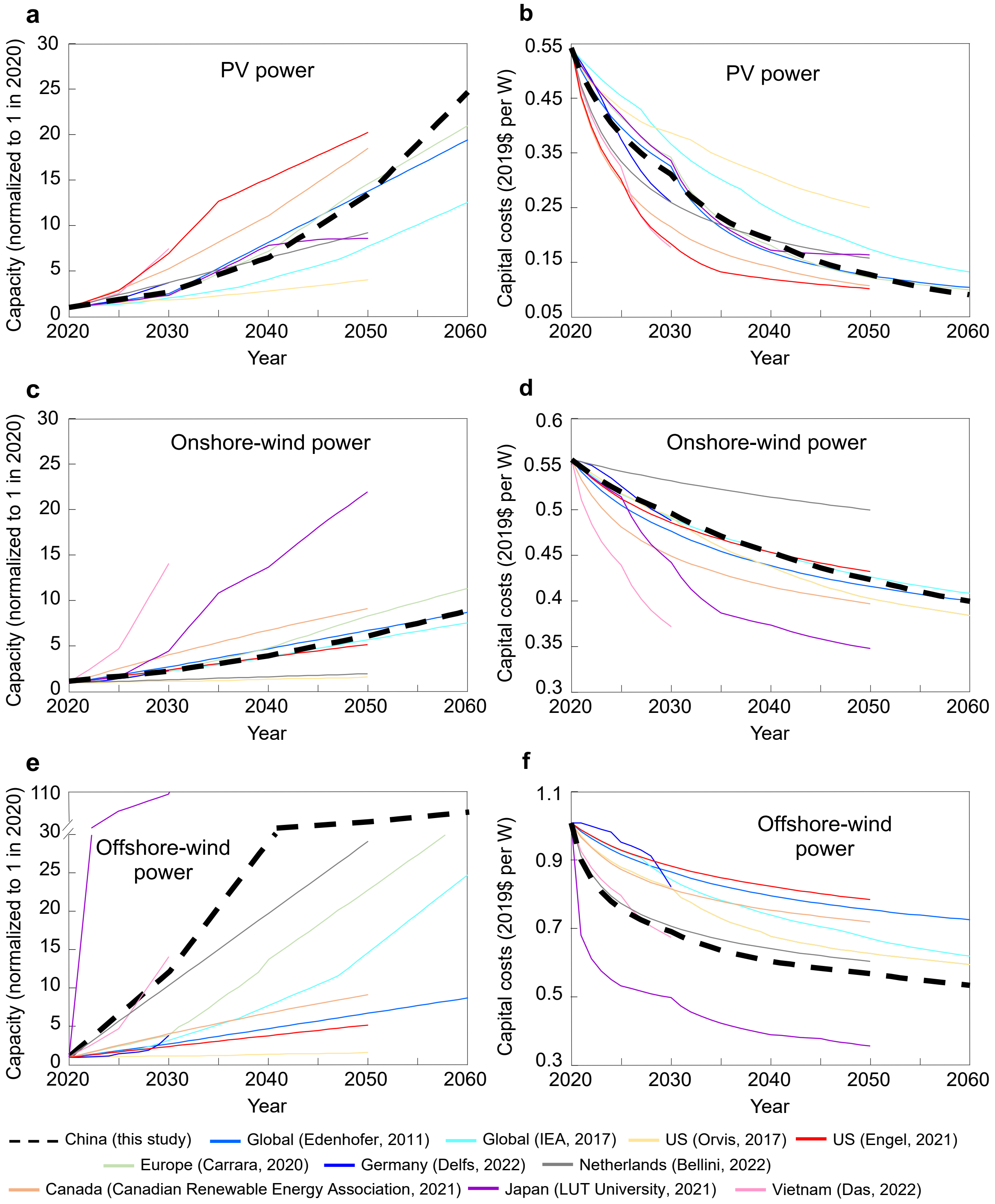

Fig. S2. Comparison of the projected growth of power capacity and the projected decline of capital costs for PV and wind-power plants in different regions. (**a, c, e**) Comparison of the projected relative increase in the installed capacity of PV (**a**), onshore-wind (**c**) and offshore-wind (**e**) power plants during 2021–2060 in China from this study, for the projected global PV and wind power estimated by the International Energy Agency (IEA) (IEA, 2017) or by Edenhofer et al. (2011), for the US in two previous studies (Orvis, 2017; Engel, 2021), for Europe in a previous study (Carrara et al., 2020), for Germany in a previous study (Delfs and Dezem, 2022), for the Netherlands in a previous study (Bellini, 2022), for Canada in a previous

study (Canadian Renewable Energy Association, 2021), for Japan in a previous study (LUT University, 2021), and for Vietnam in a previous study (Das, 2022). The installed power capacity in 2020 is normalized to one in each estimate. (**b, d, f**) The projected decline of capital costs of PV (**b**), onshore-wind (**d**) and offshore-wind (**f**) power plants due to learning during 2021–2060 based on the projected increase in the installed capacity of PV and wind-power plants in different regions.

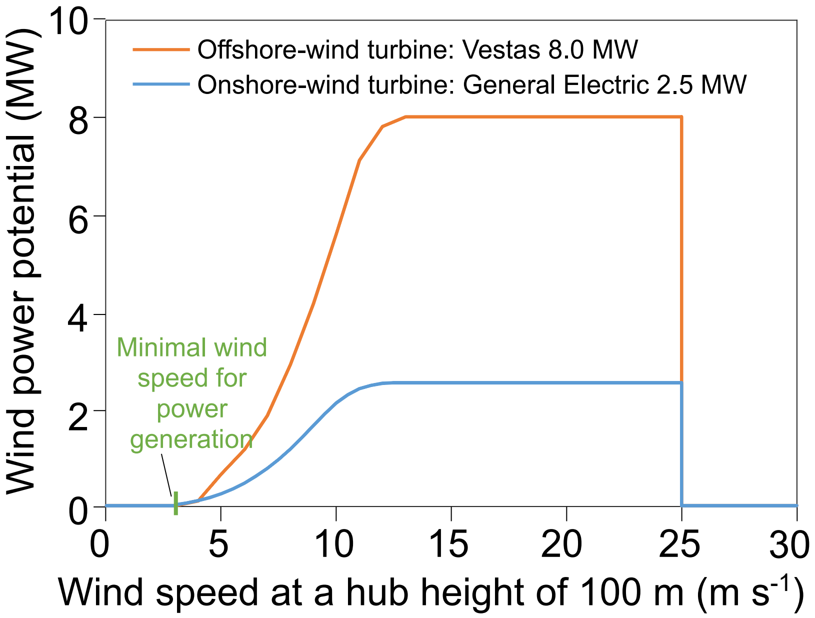

Fig. S3. **The relationship between wind speed and the wind power capacity for onshore and offshore wind turbines.** The specification of models for onshore and offshore wind turbines are General Electric (GE) 2.5 MW and Vestas 8.0 MW, respectively. The lowest and highest threshold of wind speeds at a hub height of 100 meters above ground are set at 3 and 25 m s^{-1}, respectively, to guarantee the security of wind power generation (Lu et al., 2020; Bauer and Matysik, 2021).

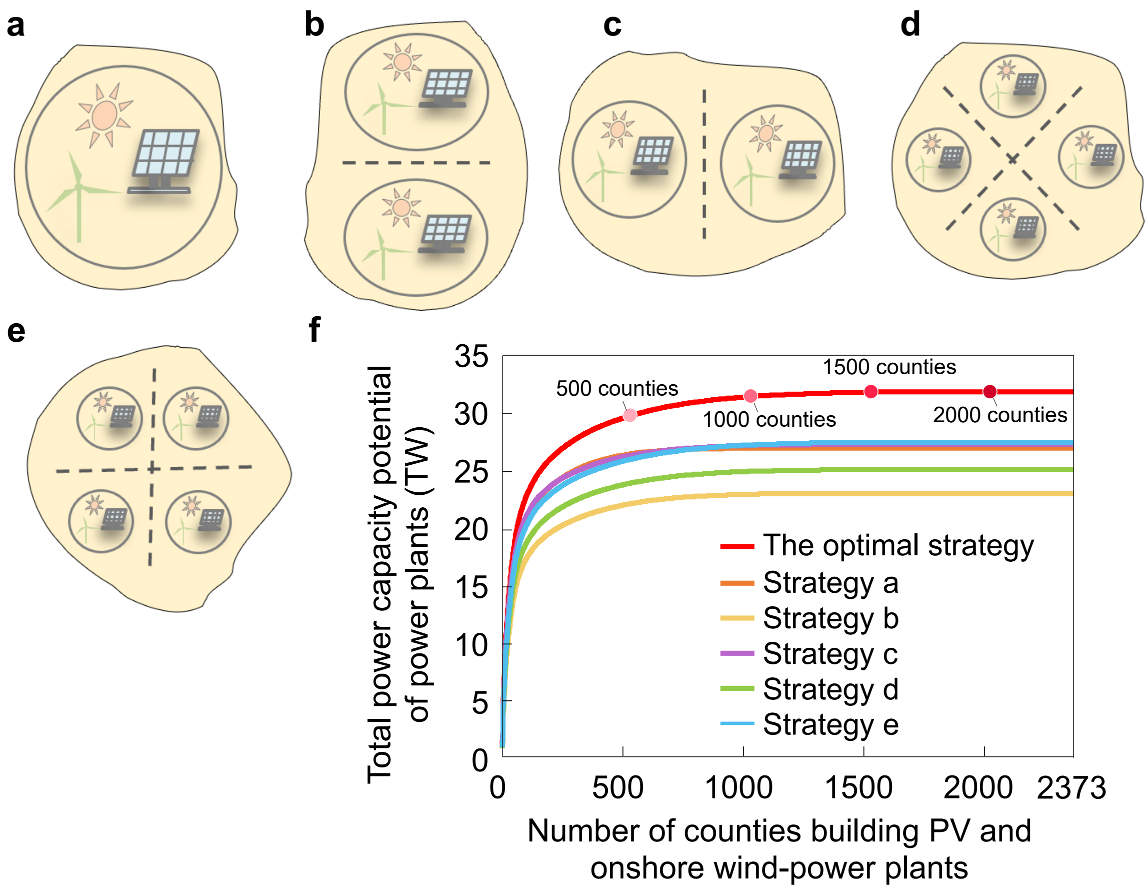

Fig. S4. Strategies of building power plants. (**a**) In the first strategy, there is only one power plant built in a county, where the center of power plant is located at the center of all pixels suitable for installing PV panels or wind turbines. (**b, c**) In the second and third strategies, there are two power plants built in a county, where the center of each power plant is located at the center of all pixels in the bottom-up (**b**) and left-right (**c**) parts. (**d, e**) In the fourth and fifth strategies, there are four power plants built in a county, where the center of each power plant is located at the center of all pixels in the east-south-west-north (**d**) and northeast-southeast-southwest-northwest (**e**) parts. (**f**) Comparison of the total power capacity adopting the best strategy of building power plants in each county with the power capacity potential when applying a strategy of building power plants in (**a–e**) for all counties.

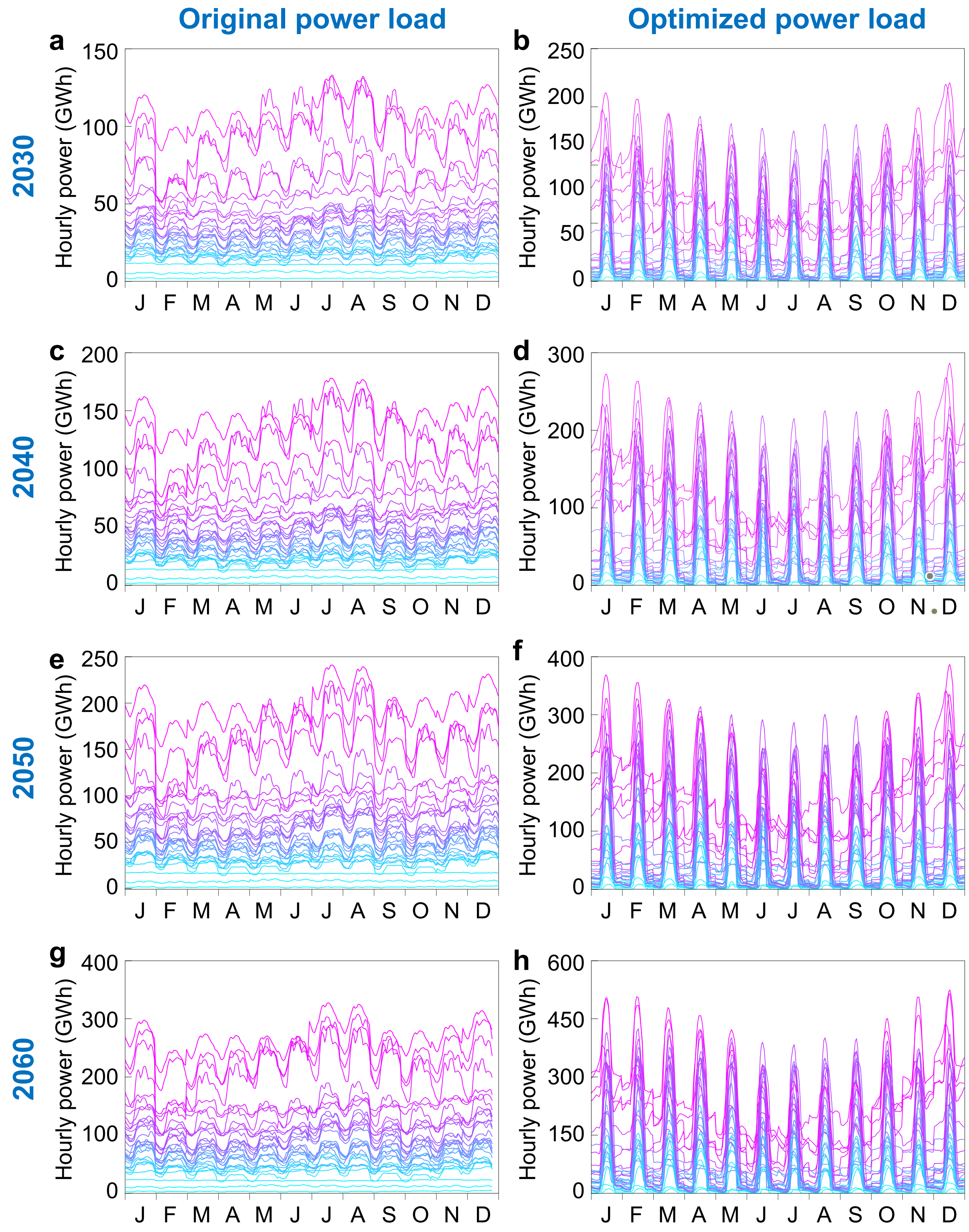

Fig. S5. Impact of optimizing the power systems on the profile of hourly power load from 2030 to 2060. Comparison between the original hourly power load (**a, c, e, g**) and the optimized flexible hourly power load to match the PV and wind power generation (**b, d, f, h**) in 2030 **(a, b)** , 2040 **(c, d)** , 2050 **(e, f)** and 2060 **(g, h)**. Each line indicates the hourly power load as an average by month in a province.

Supporting Tables

Table S1. Learning rates for photovoltaic (PV) and wind power in the literature.

Region	Time coverages	Capital costs	Learning rates	Reference(s)
Global	2006–2016	Costs of PV modules, inverters and switchgears	33.4% for 2006–2011, 27.4% for 2011–2016 and 37.1% for 2011–2016.	Lilliestam et al., 2020
Global	1976–2015	PV module costs	20.1±0.5% for 1976–2015	Louwen et al., 2016
Global	2000–2017	PV module costs	17.9% for 2000–2004, 13% for 2005–2011 and 34.9% for 2012–2018.	Sendagorta, 2019
Global	1975–2015	PV module costs	18%	Sivaram et al., 2016
Global	1975–2019	PV module costs	20%	Verlinden et al., 2013
Global	1988–2006	PV module costs	7.3% for 1988–1996, 7.9% for 1997–2001, 7.2% for 2002–2006 at current price; 15.5% for 1988–1996, 16.2% for 1997–2001 and 14.9% for 2002–2006.	Gan et al., 2015
Global	1976–1910	PV c–Si module price	30% for 1976–1988 and 17% for 1989–2010.	Kersten et al., 2011
Global	1980–2001	PV module price	21.62%	Nemet, 2006
EU	1985–1995	PV production costs (ECU per kWh)	35%	International Energy Agency, 2000
Germany	2006–2020	PV module prices	20%	Helveston et al., 2022
United States	2006–2020	PV module prices	26.40%	Helveston et al., 2022
China	2007–2020	PV module prices	32.20%	Helveston et al., 2022
China	2000–2017	PV power investment cost	22.00%	Xu al., 2020

China	2005–2009	PV installing cost	43.00%	Rigter al., 2010
Global	1980–2010	Wind turbine costs	6% for onshore wind turbines and 3% for offshore wind turbines.	Criqui et al., 2015
Global	1990–2015	Wind turbine costs	9.8% with a range of 7.7%–11%	Williams et al., 2017
Global	1985–2050	Offshore wind turbine costs	10% for 1985–2004, 2011–2030 and 5% for 2030–2050	Lemming et al., 2009
Global	1979–2010	Wind turbine costs	12% for onshore wind turbines in 1979–2010 and 12% for offshore wind turbines in 1985–2005	Rubin et al., 2015
China	——	Wind power cost	15%	Wesseh et al., 2016
China	——	Wind power cost	12.62%	Li et al., 2016
China	2004–2015	Capital costs of onshore wind power	7.5% based on 2059 onshore wind projects	Tu et al., 2019
China	2008–2012	Wind turbine costs	12%	Di et al., 2012
Germany	1990–1998	Wind turbine price	8%	Wene, 1998
Denmark	1984–1999	Wind turbine price	11.7% for 1984–1988 and 7.5% for 1988–1998	Ibenholt, 2002
Denmark	1982–1997	Wind turbine costs	8%	Neij, 2004
**Average for all data**				
China		PV modules	32.40±8.57% (std)	
International		PV modules	20.76±9.36% (std)	
China		Wind turbines	11.78±2.71% (std)	
International		Wind turbines	8.45±2.84% (std)	

Table S2. Input data for the optimisation model.

Type	Data sources
Land-cover	The land-cover data in 2019 at a spatial resolution of 0.005°×0.005° were compiled from the Moderate Resolution Imaging Spectroradiometer (MODIS) Land Cover Type 1 (MCD12Q1) data set (United States Geological Survey, 2014), which were used to estimate the suitability factor and the terrestrial carbon sink (https://lpdaac.usgs.gov/products/mcd12q1v006/).
Solar radiation	The geospatial data of hourly direct and diffuse solar radiation during 2012–2018 at a spatial resolution of 0.25° in latitude and 0.31° in longitude were compiled from NASA's Goddard Earth Observing System Model, version 5 (GEOS-5) Forward Processing (FP) data set (Global Modeling and Assimilation Office, 2021), which were used to identify the pixels suitable for installing PV panels and estimate the power generation potential of PV energy (https://portal.nccs.nasa.gov/cgi-lats4d/opendap.cgi?&path=GEOS-5/fp/0.25_deg/assim).
Wind speed at a hub height of 100 meters above ground (V)	The hourly wind speed at a height of 100 meters above ground at a spatial resolution of 0.5° in latitude and 0.625° in longitude during 2012–2018 were calculated based on the hourly friction velocity, the displacement length and the roughness length from the Modern-Era Retrospective analysis for Research and Applications, version 2 (MERRA-2) data set (Gelaro et al., 2017) (https://portal.nccs.nasa.gov/cgi-lats4d/opendap.cgi?&path=GEOS-5/fp/0.25_deg/assim).
Air temperature at 2 meters (T_{atm})	The geospatial data of hourly air temperature at 2 meters above ground at a spatial resolution of 0.25° in latitude and 0.31° in longitude during 2012–2018 were compiled from the NASA's Goddard Earth Observing System Model, version 5 (GEOS-5) Forward Processing (FP) data set (Global Modeling and Assimilation Office, 2021), which were adopted to identify the pixels suitable for installing PV panels and estimate the power generation potential of PV energy (https://portal.nccs.nasa.gov/cgi-lats4d/opendap.cgi?&path=GEOS-5/fp/0.25_deg/assim).
Ground slope	Ground slope data at a spatial resolution of 0.001°×0.001° observed in 2000 were compiled from the Shuttle Radar Topography Mission (SRTM) global enhanced slope data set (United States Geological Survey, 2015), which were used to identify the pixels suitable for installing PV panels and onshore wind turbines (https://lpdaac.usgs.gov/products/srtmgl1v003/)
Mask of Territorial Sea Area (TSA)	Territorial Sea Area in 2018 were compiled from the Maritime Boundaries Geodatabase (Flanders Marine Institute, 2018) to identify the pixels suitable for installing offshore wind turbines (http://www.vliz.be/en/imis?dasid=5465&doiid=312).
Mask of natural reserves	Mask of terrestrial ecological reserve in 2020 at a spatial resolution of 0.001°×0.001° were derived from the Resource and Environment Science and Data Center (Resource and Environment Science and Data Center, 2020) (http://www.resdc.cn/data.aspx?DATAID=137), and the mask of marine ecological reserve in 2021 were derived from the National Marine Data and Information Service (UN Environment Programme World Conservation Monitoring Centre, 2021; Resource and Environment Science and Data Center, 2020), which were adopted to identify the pixels suitable for installing PV panels,

	onshore wind turbines and offshore wind turbines (https://www.protectedplanet.net/country/CHN).
Water depth (D_P)	The geospatial data of water depth at a spatial resolution of 0.001°×0.001° observed in 2000 were compiled from the Radar Topography Mission (SRTM) Global Enhanced Slope (GES) data set (United States Geological Survey, 2015) to identify the pixels suitable for installing offshore wind turbines (https://lpdaac.usgs.gov/products/srtmgl1v003/).
Shipping routes	The geospatial data of sulfur dioxide (SO_2) emission rates during 2012–2018 were compiled from the MERRA-2 data set (Gelaro et al., 2017) to identify the shipping routes in pixels where the annual average SO_2 emission rate is higher than 10^{-11} kg m^{-2} s^{-1} using the method in the literature (Lu et al., 2020) (https://disc.gsfc.nasa.gov/datasets/M2T1NXADG_5.12.4/summary?keywords=SO2).
Friction velocity ($u*$), zero-plane displacement height (d), and surface roughness (z_0)	The geospatial data of hourly friction velocity, zero-plane displacement height and the surface roughness during 2012–2018 at a spatial resolution of 0.5°×0.625° were compiled from the NASA's Goddard Earth Observing System Model, version 5 (GEOS-5) Forward Processing (FP) database (Global Modeling and Assimilation Office, 2021), which were used to estimate the wind speed at the hub height of 100 meters above ground (https://portal.nccs.nasa.gov/cgi-lats4d/opendap.cgi?&path=GEOS-5/fp/0.25_deg/assim).
Capacity factor of wind turbines ($CF_{onshore}$ and $CF_{offshore}$)	The capacity factor of onshore and offshore wind turbines as a function of the wind speed was predicted using the wind turbine models available at https://en.wind-turbine-models.com/turbines (Bauer and Matysik, 2021).
Terrestrial carbon sink (γ_x)	The geospatial data of terrestrial carbon sink were obtained by performing a disaggregation of the total net terrestrial carbon sink uptake in China (1.87 Gt CO_2 y^{-1}) inferred from measurements of atmospheric CO_2 gradients from 2006–2009 (Jiang et al., 2016) using the geospatial data of terrestrial carbon sink at a spatial resolution of 4° in latitude and 5° in longitude from 2010–2016 (Wang et al., 2020) as a proxy.
Energy consumption by province	Consumption of primary energy and electricity by province in the power, residential, industrial, transportation and other sectors in 2019 were compiled from the Chinese Energy Statistical Yearbook 2020 (National Bureau of Statistics of China, 2020).
Spatial distribution of GDP	The spatial distribution of GDP in 2015 at a spatial resolution of 0.01°×0.01° were compiled from the Resource and Environment Science and Data Center (Xu, 2017) (https://www.resdc.cn/data.aspx?DATAID=252).
Annual mean temperature change	The change in annual mean surface air temperature during 2021–2060 for China were derived from simulations in the SSP1-2.6 scenario in an Earth system model (Gasser, 2017).
Traffic flow data	The profile of traffic flow was derived from observations in each street per 5 minutes in 2018 in Shenzhen as a megacity in China (Shenzhen Municipal Government data open platform, 2018)
Historical	The typical power load profiles by month in each province in 2018 were compiled

power load profiles	from the National Development and Reform Commission database (National Development and Reform Commission, 2019).
Power generation by oil, gas, bioenergy, nuclear and hydropower	Power generation by nuclear, hydro, hydrogen and bioenergy in 2019 was compiled by province from National Statistical Yearbook of Energy (National Bureau of Statistics of China, 2020). The power generation by oil, gas, bioenergy, nuclear and hydropower were derived as the average of three IAMs (SWITCH, IPAC and GCAM_TU) in the "1.5℃-limiting" scenario for 2021–2060 from a mutli-model study (Duan, 2021).
Resident income for urban and rural populations	Per capita disposable income for urban and rural populations in 2,373 counties in China in 2019 were compiled from the Chinese Statistical Yearbook (National Bureau of Statistics of China, 2020) (https://data.cnki.net/Yearbook/Navi?type=type&code=A).
Income distribution for urban and rural populations	The residents' income distribution for urban and rural populations were compiled from the national socioeconomic survey in 2015 (Zhang et al., 2016).

Table S3. Indicators used to filter the pixels suitable for installing PV panels or wind turbines.

Indicators		PV	Onshore wind	Offshore wind
Suitability factor of land-cover (%)	Forests	0	0	0
	Closed Shrubland	15	100	0
	Open Shrubland	15	100	0
	Woody savanna	15	100	0
	Savanna	15	100	0
	Grassland	15	100	0
	Wetland	0	0	0
	Croplands	0	100	0
	Urban	0	0	0
	Vegetation mosaics	0	100	0
	Snow, ice	10	0	0
	Deserts	15	100	0
	Water bodies	0	0	100
Slope (%)		<5	<20	—
Solar radiation (hour per d)		>4.2	—	—
Annual average capacity factor (%)		—	>20	—
Surface air temperature (℃)		> 0	—	—
Altitude above sea level (m)		—	<3,000	—
Water depth in the ocean (m)		—	—	≤60
Natural reserves		Excluding terrestrial and marine reserves		
Mask of territorial sea		—	—	Within China's Exclusive Economic Zone
Shipping routes		—	—	Excluding 20% area of pixels with the emission rate of SO_2 above 10^{-11} kg m^{-2} s^{-1}

Table S4. Configurations of the onshore and offshore wind turbines.

**Parameters**	**General Electric 2.5 MW (onshore wind turbines)**	**Vestas 8.0 MW (offshore wind turbines)**
Rated power	2,500 kW	8,000 kW
Cut-in wind speed	3 m s^{-1}	3 m s^{-1}
Rated wind speed	12 m s^{-1}	13 m s^{-1}
Cut-out wind speed	25 m s^{-1}	25 m s^{-1}
Diameter	103 m	164 m
Swept area	8,332 m^2	21,124 m^2
Number of blades	3	3
Hub height	100 m	100 m

Table S5. Parameters of the pumped-hydro storage and chemical battery storage.

Parameters		Pumped-hydro storage	Chemical battery storage	References
Lifetime (years)		50	15	Cole and Frazier, 2019; Chen et al., 2021
Round-trip efficiency (%)		70%	85%	Cole and Frazier, 2019; Chen et al., 2021
Energy loss rate (%)		0%	1%	Xiong and Singh, 2015
Per unit throughput (kWh per kWh)		N/A	6,000	Chen et al., 2021
Minimum/Maximum residual energy rate (%)		100%/0%	100%/0%	Chen et al., 2021
Energy-specific costs (2019$ per kWh)	2020	100	345	Cole and Frazier, 2019; Chen et al., 2021
	2030	100	198	
	2040	100	174	
	2050	100	149	
	2060	100	124	
Power-specific costs (2019$ per kW)	2020	1,200	595.73	Cole and Frazier, 2019; Hiesl et al., 2020
	2030	1,200	374.45	
	2040	1,200	327.22	
	2050	1,200	280.78	
	2060	1,200	234.34	
Optional costs (2019$ per kWh)		0.0015	0.0015	Zhang et al., 2016

Table S6. Parameters used to estimate the power generation by photovoltaic (PV), and wind-power plants.

Symbols	Parameters	Values	References
k	Von Karman constant	0.41	Rinne et al., 2018
SR	Maximal power for PV panels	161.9 Watt per m^2	Chen et al., 2019
r_{loss}	Electricity loss of PV power generation due to grid connection	19.44%	Chen et al., 2019
T_{cell}	Normal cell operating temperature	44 °C	Chen et al., 2019
σ_T	Temperature coefficient	–0.41 % per °C	Chen et al., 2019
$PW_{onshore}$	Maximal power of onshore wind turbine	2.5 MW	Lu et al., 2020
$D_{onshore}$	Diameter of rotor for onshore wind turbines	103 meters	Bauer and Matysik, 2021
$PW_{offshore}$	Maximal power of offshore wind turbine	8 MW	Lu et al., 2020
$D_{offshore}$	Diameter of rotor for offshore wind turbines	164 meters	Bauer and Matysik, 2021
U_{TI}	Efficiency of energy conversion by wind turbines	95%	Rinne et al., 2018
A_{RR}	Array efficiency factor of wind turbines	90%	Rinne et al., 2018
F_{EN}	Electricity loss of offshore wind power generation due to the environmental and curtailment effects	2%	Musial et al., 2016

Table S7. Parameters used to estimate the costs of photovoltaic (PV) and wind power generation.

Symbols	Parameters	Values (Average ± standard deviation)	References
μ_{module}	Module costs	\$0.25±0.03 per Watt for PV panels and \$0.39±0.04 per Watt for wind turbines	China Photovoltaic Industry Alliance, 2021; Liu et al., 2015b
$\mu_{inverter}$	Inverter costs	\$0.02±0.002 per Watt for PV panels	China Photovoltaic Industry Alliance, 2021
μ_{ins}	Installation work costs	\$0.11±0.01 per Watt for PV panels and \$0.12±0.01 per Watt for wind turbines	China Photovoltaic Industry Alliance, 2021; Liu et al., 2015b
μ_{adm}	Administration costs	\$0.04±0.004 per Watt for PV panels and \$0.02±0.002 per Watt for wind turbines	China Photovoltaic Industry Alliance, 2021; Liu et al., 2015b
μ_{grid}	Grid connection costs	\$0.03±0.003 per Watt for PV panels and \$0.02±0.002 per Watt for wind turbines	China Photovoltaic Industry Alliance, 2021; Liu et al., 2015b
μ_{sec}	Secondary equipment costs	\$0.01±0.001 per Watt for PV panels	China Photovoltaic Industry Alliance, 2021
$\mu_{mounting}$	Mounting materials costs	\$0.05±0.005 per Watt for PV panels	China Photovoltaic Industry Alliance, 2021
μ_{land}	Unit costs of land acquisition	\$16.83±1.68, \$30.91±3.09, \$30.46±3.05 and \$4.69±0.47 per m^2 for cropland, forest, built-up lands and barren, respectively.	Ministry of Land and Resources, 2015; China Renewable Energy Engineering Institute (CREEI) et al., 2012
μ_{line}	Unit costs of lines for PV panel or wind turbine connection	\$388,000±38,800 per km	Electric Power Planning and Engineering Institute, 2020
μ_{tran}	Unit costs of voltage transformator	\$39671,100±3967,110 per 300MW	Electric Power Planning and Engineering Institute, 2020
$\mu_{baseline}$	Unit costs of capacity potential for a standard offshore wind turbine	\$2,000±200 per kW	Sherman et al., 2017

R_y	The ratio of O&M costs to initial investment costs	1%, 3% and 3% for PV, onshore-wind and offshore-wind power plants, respectively	Yan et al., 2019; Xiang et al., 2021
r	The discounting rate	5% per y	Duan et al., 2021
θ_{fossil}	CO_2 emission factor of fossil fuels	0.84, 0.72 and 0.46 kg CO_2 per kWh for coal, oil and gas, respectively	Liu et al., 2015a; National Bureau of Statistics of China, 2020
P_0	Initial capacities in 2020	253,000 MW for PV power, 272,010 MW for onshore wind power and 8,990 MW for offshore wind power	China Energy Storage Network, 2021
r_{LR}	Learning rates	PV modules (**Supplementary Table 1**)	
		Wind turbines (**Supplementary Table 1**)	
		18%±5% for inverters, mounting materials, secondary equipment, installation work and administration and grid connection	Sweerts et al., 2020
		18%±5% for power transmission	Sweerts et al., 2020

Table S8. Parameters for ultra-high-voltage (UHV) transmission and energy storages.

Symbols	Parameters	Values (Average ± standard deviation)	References
μ_{lines}	Line cost per kilometer	$732,220±73,222 per km for ±800 kV DC<br><br>$800,383±80,038 per km for ±1,100 kV DC<br><br>$670,785±67,079 per km for 1,000 kV AC	Electric Power Planning and Engineering Institute, 2011
μ_{opera}	Unit operational costs in charging and discharging	$0.0015±0.0001 per kWh	Zhang et al., 2016
μ_{sub}	Unit costs of converters for DC and substations for AC	$82±8.2 per kW for ±800 kV DC<br><br>$92±9.2 per kW for ±1,100 kV DC<br><br>$41±4.1 per kW for 1,000 kV AC	Electric Power Planning and Engineering Institute, 2011
μ_{power}	Unit costs of energy storage	$1,200±120 per kW for pumped-hydro storage in 2020–2060	Hiesl et al., 2020
		$595±60 per kW, $374±37 per kW, $327±33 per kW, $280±28 per kW and $234±23 per kW for lithium battery storage in 2020, 2030, 2040, 2050 and 2060, respectively	Cole and Frazier, 2019
μ_{throu}	Unit cost of throughput in the energy storages	$100±10 per kWh for pumped-hydro storage in 2020–2060	Chen et al., 2021
		$345±34 per kWh, $198±20 per kWh, $174±17 per kWh, $149±15 per kWh and $124±12 per kWh for lithium battery storage in 2020, 2030, 2040, 2050 and 2060, respectively	Cole and Frazier, 2019
ϱ	Prices of fossil fuel	$0.043±0.015 per kWh for coal	Statista, 2011
		$0.058±0.016 per kWh for gas	National Development and Reform Commission, 2019
		$0.141±0.057 per kWh for oil	Yte1, 2022; China National Petroleum

			Corp, 2012
N_c	The number of annual charging and discharging for storage system	365 and 6,000/15 for pumped-hydro storage and lithium battery storage, respectively	Chen et al., 2021; Cole and Frazier, 2019
ε	The ratio of residual energy after charging and discharging	70% for pumped-hydro storage	Chen et al., 2021
		85% for lithium battery storage	Cole and Frazier, 2019;
P_{UHV}	Capacity of each transmission line	8,000 MW for ±800 kV DC and 12,000 MW for ±1,100kV DC. For 1,000 kV AC, the capacity is a function of the length of line, which decreases from 6,000 MW for 100 km to 3,000 MW for 3,000 km.	Chen et al., 2021

Table S9. Soil carbon content by zone in China. The data are compiled from a national field survey (Lai et al., 2016).

Vegetation	Zone	Carbon content (tC per ha)
Evergreen Needleleaf Forests	Temperate	51.8
	Subtropics	22.1
	Tropics	22.1
Evergreen Broadleaf Forests	Subtropics	50.8
	Tropics	68.2
Deciduous Needleleaf Forests	All	52.3
Deciduous Broadleaf Forests	All	35.5
Mixed Forests	Temperate	48.0
	Subtropics	50.6
Closed Shrublands<br>Open Shrublands	Plateau-climate	3.3
	Temperate	6.95
	Subtropics	10.94
	Tropics	10.225
Woody Savannas	Plateau-climate	1.8
	Temperate, Subtropics and Tropics	3.7
Savannas	Plateau-climate	1.8
	Temperate, Subtropics and Tropics	3.7
Grasslands	Plateau-climate	1.8
	Temperate, Subtropics and Tropics	2.4
Permanent Wetlands	All	3.9
Croplands	All	5.7

Urban and Built-up Lands	All	0.0
Cropland/Natural Vegetation Mosaics	All	5.7
Permanent Snow and Ice	All	0.0
Desert	All	0.0
Water Bodies	All	0.0
Unclassified	All	0.0

427

Table S10. Projection of hourly power demand in different sectors by 2060.

Type	Sectors	Methods
**Prescribed hourly power load under a warming climate**	Cooling and Space heating	The hourly power load during 2021–2060 was predicted based on the projected increase in power demand in the residential sector under an electrification rate of 58% by 2060 (International Energy Agency, 2021), the hourly gridded temperature in 2020 compiled from the Goddard Earth Observing System Model (https://gmao.gsfc.nasa.gov/GMAO_products/NRT_products.php) and the change in annual mean temperature during 2021–2060 over the China region under the SSP1-2.6 scenario simulated by an Earth system model (Gasser et al., 2017).
	Transportation	The hourly power load during 2021–2060 was predicted based on the projected increase in power demand in the transportation sector under an electrification rate of 58% (International Energy Agency, 2021), the hourly traffic flow data in Shenzhen city in 2018, the hourly gridded temperature in 2020 compiled from the Goddard Earth Observing System Model (https://gmao.gsfc.nasa.gov/GMAO_products/NRT_products.php) and the change in annual mean temperature during 2021–2060 over the China region under the SSP1-2.6 scenario simulated by an Earth system model (Gasser et al., 2017).
**Endogenous hourly power load to match the power generation by PV and wind-power plants**	Agriculture	The hourly power load during 2021–2060 is simulated endogenously by decade when the hourly power load profile is adjusted to match the hourly power generation by PV and wind power when meeting different targets of CO_2 emission abatements. To obtain the hourly power load before this optimisation, the historical hourly power load profile from the provincial electrical grids in 2018 (National Development and Reform Commission, 2019) was scaled up by the projected increase in power demand during 2021–2060 under an electrification rate of 58% (International Energy Agency, 2021).
	Industry	
	Building	
	Services	
	Household electric appliance except for heating and cooling	
	Others	

Supporting references

1. Bauer, L. & Matysik, S. Wind Turbine Specification. Available at: https://en.wind-turbine-models.com/turbines (2021).
2. Becker, J. J. *et al*. Global bathymetry and elevation data at 30 arc seconds resolution: SRTM30_PLUS. *Mar. Geod.* **32**, 355–371 (2009).
3. Bellini, E. Netherlands may reach 132 GW of solar by 2050. Available at: https://www.pv-magazine.com/2022/04/22/netherlands-may-reach-135-gw-of-solar-by-2050/ (2022).
4. Canadian Renewable Energy Association. Powering Canada's Journey to Net-Zero: CanREA's 2050 Vision. Available at: https://renewablesassociation.ca/wp-content/uploads/2021/11/CanREAs2050Vision_Nov2021_web.pdf (2021).
5. Carrara, S. *et al.* Raw materials demand for wind and solar PV technologies in the transition towards a decarbonised energy system. Luxembourg: Publications Office of the European Union. Available at: https://eitrawmaterials.eu/wp-content/uploads/2020/04/rms_for_wind_and_solar_published_v2.pdf (2020).
6. Chen, S. *et al*. The potential of photovoltaics to power the belt and road initiative. *Joule* **3**, 1895–1912 (2019).
7. Chen, X. *et al*. Pathway toward carbon-neutral electrical systems in China by mid-century with negative CO_2 abatement costs informed by high-resolution modeling. *Joule* **5**, 2715–2741 (2021).
8. China Electricity Council. Main indicators of national economy in 2020. Available at: https://www.cec.org.cn/upload/1/editor/1640595481946.pdf (2021).
9. China Energy Storage Network. *A list of details of photovoltaic, wind power installed capacity and power generation capacity in each province in 2020*. Available at: http://escn.com.cn/news/show-1176657.html (2021).
10. China National Petroleum Corp. Domestic fuel oil market annual Analysis report in 2011. Available at: https://view.officeapps.live.com/op/view.aspx?src=http%3A%2F%2Foilinfo.cnpc.com.cn%2Fypxx%2Fyjnb%2F201208%2F24404fbc0eed4f2280939eae3c4ea8ad%2Ffiles%2F8920787449ee417997c0147be45fce98.doc&wdOrigin=BROWSELINK (2012).
11. China Photovoltaic Industry Alliance. *China PV industry development roadmap (Version 2020)*. Available at: http://chinapv.org.cn/road_map/927.html (2021).
12. China Renewable Energy Engineering Institute. *Construction Land Index of Power Engineering Project - Wind Farm*. (China Electric Power Press, Beijing, 2012).
13. Cole, W. J. & Frazier, A. *Cost projections for utility-scale battery storage*. Report number: NREL/TP-6A20-73222. (National Renewable Energy Lab, Golden, CO, United States, 2019).

14. Criqui, P., Mima, S., Menanteau, P. & Kitous, A. Mitigation strategies and energy technology learning: An assessment with the POLES model. *Technol. Forecast. Soc. Change* **90**, 119–136 (2015).

15. Das, K. Renewables in Vietnam: Current Opportunities and Future Outlook. Available at: https://www.vietnam-briefing.com/news/vietnams-push-for-renewable-energy.html/ (2020).

16. Delfs, A. & Dezem, V. Germany Brings Forward Goal of 100% Renewable Power to 2035. Available at: https://www.bloomberg.com/news/articles/2022-02-28/germany-brings-forward-goal-of-100-renewable-energy-to-2035?leadSource=uverify%20wall (2022).

17. Di, Y., Cui, X. & Liu, X. The impact of technology innovations on cost of China's wind power industry. *J. Quan. Tech. Econ.* **3**, 140–150 (2012).

18. Duan, H. *et al*. Assessing China's efforts to pursue the 1.5° C warming limit. *Science* **372**, 378–385 (2021).

19. Dunnett, S., Sorichetta, A., Taylor, G. & Eigenbrod, F. Harmonised global datasets of wind and solar farm locations and power. *Sci. Data* **7**, 1–12 (2020).

20. Edenhofer, O. *et al.* Renewable energy sources and climate change mitigation: Special report of the intergovernmental panel on climate change. Cambridge University Press. Available at: https://www.ipcc.ch/site/assets/uploads/2018/03/SRREN_Full_Report-1.pdf (2011).

21. Electric Power Planning and Engineering Institute. *Power grid engineering quota design control index*. (China Electric Power Press, Beijing, 2020).

22. Electric Power Planning and Engineering Institute. *The Grid Project Construction Cost Analysis in the 11th Five-year Period.* (China Electric Power Press, Beijing, 2011).

23. Engel, J. Biden blueprint calls for wind and solar to power 90% of U.S. grid by 2050. Available at: https://www.renewableenergyworld.com/solar/biden-blueprint-calls-for-wind-and-solar-to-power-90-of-u-s-grid-by-2050/#gref (2021).

24. Flanders Marine Institute. *Maritime boundaries geodatabase: Maritime boundaries and exclusive economic zones (200NM)*. Available at: http://www.vliz.be/en/imis?dasid=5465&doiid=312 (2018).

25. Gan, P. & Li, Z. Quantitative study on long term global solar photovoltaic market. *Renew. Sust. Energ. Rev.* **46**, 88–99 (2015).

26. Gasser, T. *et al*. The compact Earth system model OSCAR v2.2: description and first results. *Geosci. Model Dev*. **10**, 271–319 (2017).

27. Gelaro, R. *et al*. The modern-era retrospective analysis for research and applications, version 2 (MERRA-2). *J. Climate* **30**, 5419–5454 (2017)

28. Center for Security and Emerging Technology. Translation: Outline of the People's Republic of China 14th Five-Year Plan for National Economic and Social Development and Long-Range Objectives for 2035. Available at: https://cset.georgetown.edu/publication/china-14th-five-year-plan/ (2021).
29. Global Modeling and Assimilation Office. *GEOS Atmospheric Assimilation Products.* Available at: https://gmao.gsfc.nasa.gov/GMAO_products/NRT_products.php (2021).
30. Helveston, J. P., He, G. & Davidson, M. R. Quantifying the cost savings of global solar photovoltaic supply chains. *Nature* **612**, 83–87 (2022).
31. Hiesl, A., Ajanovic, A. & Haas, R. On current and future economics of electricity storage. *Greenh. Gases*. **10**, 176–1192 (2020).
32. Ibenholt, K. Explaining learning curves for wind power. *Energy Policy* **30**, 1181–1189 (2002).
33. Imamovic, A., Tanaka, K., Folini, D., & Wild, M. Global dimming and urbanization: did stronger negative SSR trends collocate with regions of population growth? *Atmospheric Chem. Phys.* **16**, 2719–2725 (2016).
34. International Energy Agency. *An Energy Sector Roadmap to Carbon Neutrality in China*. (International Energy Agency, Paris, France, 2021).
35. International Energy Agency. *Energy technology perspectives 2017: Catalysing Energy Technology Transformations* (2017).
36. International Energy Agency. *Experience curves for energy technology policy*. (International Energy Agency, Paris, France, 2000).
37. Jacobson, M. Z. & Jadhav, V. World estimates of PV optimal tilt angles and ratios of sunlight incident upon tilted and tracked PV panels relative to horizontal panels. *Sol. Energy* **69**, 55–66 (2018).
38. Jiang, F. *et al*. A comprehensive estimate of recent carbon sinks in China using both top-down and bottom-up approaches. *Sci. Rep.* **6**, 22130 (2016).
39. Kaldellis, J. K., Kapsali, M. & Kavadias, K. A. Temperature and wind speed impact on the efficiency of PV installations. Experience obtained from outdoor measurements in Greece. *Renew. Energ.* **66**, 612–624 (2014).
40. Kawajiri, K., Oozeki, T. & Genchi, Y. Effect of temperature on PV potential in the world. *Environ. Sci. Technol.* **45**, 9030–9035 (2011).
41. Kersten, F. *et al. PV learning curves: past and future drivers of cost reduction.* (Proceedings of the 26th EU PVSEC conference, Hamburg, 2011).
42. Lai, L. *et al*. Carbon emissions from land-use change and management in China between 1990 and 2010. *Sci. Adv.* **2**, e1601063 (2016).
43. Lemming, J. K., Morthorst, P. E. & Clausen, N. E. Offshore Wind Power Experiences, Potential and Key Issues for Deployment. *Forskningscenter Risø Roskilde* (2009).

44. Li, H. *et al*. Could wind and PV energies achieve the grid parity in China until 2020?. *Filomat* **30**, 4173–4189 (2016).
45. Lilliestam, J., Melliger, M., Ollier, L., Schmidt, T. S. & Steffen, B. Understanding and accounting for the effect of exchange rate fluctuations on global learning rates. *Nat. Energy* **5**, 71–78 (2020).
46. Liu, Z. *et al*. Reduced carbon emission estimates from fossil fuel combustion and cement production in China. *Nature* **524**, 335–338 (2015a).
47. Liu, Z., Zhang, W., Zhao, C. & Yuan, J. The economics of wind power in China and policy implications. *Energy* **8**, 1529–1546 (2015b).
48. Lorenczik, S. *et al*. *Projected costs of generating electricity-2020 edition.* (Organisation for Economic Co-Operation and Development, Paris, France, 2020).
49. Louwen, A., Van Sark, W. G., Faaij, A. P. & Schropp, R. E. Re-assessment of net energy production and greenhouse gas emissions avoidance after 40 years of photovoltaics development. *Nat. Commun.* **7**, 1–9 (2016).
50. Lu, T. *et al*. India's potential for integrating solar and on-and offshore wind power into its energy system. *Nat. Commun.* **11**, 1–10 (2020).
51. LUT University. Renewable pathways to climate-neutral Japan. Available at: https://static.agora-energiewende.de/fileadmin/Projekte/2021/2021_03_JP_2050_study/2021_LUT-Agora-REI_Renewable_pathways_Summary.pdf (2021).
52. Masters, G. M. Renewable and Efficient Electric Power Systems. (*John Wiley & Sons*, 2013).
53. Mcdonald, A. & Schrattenholzer, L. Learning rates for energy technologies. *Energy Policy* **29**, 255–261 (2001).
54. Mi, Z. *et al*. Economic development and converging household carbon footprints in China. *Nat. Sustain.* **3**, 529–537 (2020).
55. Ministry of Land and Resources. *Rural Land Compensation Policy Document of Ministry of Land and Resources*. (2015).
56. Moseid, K. O. *et al.* Bias in CMIP6 models as compared to observed regional dimming and brightening. *Atmos. Chem. Phys.* **20**, 16023–16040 (2020).
57. Musial, W., Heimiller, D., Beiter, P., Scott, G. & Draxl, C. 2016 offshore wind energy resource assessment for the United States. (National Renewable Energy Lab, Golden, CO, United States, 2016).
58. National Bureau of Statistics of China. *China Energy Statistical Yearbook 2020*. (China Statistics Press, Beijing, 2020).

59. National Development and Reform Commission. Price list of benchmark gate stations for non-residential natural gas in each province. Available at: http://www.gov.cn/xinwen/2019-03/29/content_5378081.htm (2019).
60. National Development and Reform Commission. *Typical Power load curves of provincial power networks in China* (2019).
61. Neij, L., Andersen, P. D. & Durstewitz, M. Experience curves for wind power. *Inter. J. Energ. Technol. Polic.* **2**, 15–32 (2004).
62. Nemet, G. F. Beyond the learning curve: factors influencing cost reductions in photovoltaics. *Energy Polic*y **34**, 3218–3232 (2006).
63. Orvis, R. America's Renewable Electricity Forecast Grows To 2050, Even Under Trump. Available at: https://www.forbes.com/sites/energyinnovation/2017/05/10/americas-renewable-electricity-forecast-grows-to-2050-even-under-trump/?sh=13ed051c16e4 (2017).
64. Resource and Environment Science and Data Center. Environmental Protection Areas. Available at: https://www.resdc.cn/data.aspx?DATAID=137 (2020).
65. Rigter, J. & Vidican, G. Cost and optimal feed-in tariff for small scale photovoltaic systems in China. *Energy Policy* **38**, 6989–7000 (2010).
66. Rinne, E., Holttinen, H., Kiviluoma, J. & Rissanen, S. Effects of turbine technology and land use on wind power resource potential. *Nat. Energy* **3**, 494–500 (2018).
67. Rubin, E. S., Azevedo, I. M., Jaramillo, P. & Yeh, S. A review of learning rates for electricity supply technologies. *Energy Policy* **86**, 198–218 (2015).
68. Sendagorta, P. L. Learning Curves Analysis for Solar PV. (Universidad Pontificia Comillas, Spanish, 2019).
69. Shenzhen Municipal Government data open platform. Street real-time data. Available at: https://opendata.sz.gov.cn/data/dataSet/toDataDetails/29200_00403589 (2018).
70. Sherman, P., Chen, X. & McElroy, M. B. Wind-generated electricity in China: Decreasing potential, inter-annual variability and association with changing climate. *Sci. Rep.* **7**, 1–10 (2017).
71. Sivaram, V. & Kann, S. Solar power needs a more ambitious cost target. *Nat. Energy* **1**, 1–3 (2016).
72. Statista. China Qinhuangdao coal spot price from 2003 to 2019. https://www.statista.com/statistics/383534/asian-coal-marker-price/ (2021).
73. Sweerts, B., Detz, R. J. & van der Zwaan, B. Evaluating the role of Unit Size in learning-by-doing of energy technologies. *Joule* **4**, 967–970 (2020).
74. Tu, Q., Betz, R., Mo, J., Fan, Y. & Liu, Y. Achieving grid parity of wind power in China - Present levelized cost of electricity and future evolution. *Appl. Energy* **250**, 1053–1064

(2019).
75. UN Environment Programme World Conservation Monitoring Centre. Protected Area Profile for China from the World Database of Protected Areas. Available at: www.protectedplanet.net/ (2021).
76. United States Geological Survey. Land cover type yearly L3 global 500m SIN grid. Available at: https://lpdaac.usgs.gov/products/mcd12q1v006/ (2014).
77. United States Geological Survey. *Shuttle radar topography mission (SRTM)*. Available at: https://earthexplorer.usgs.gov/ (2015).
78. Verlinden, P., Zhang, Y. & Feng, Z. Cost analysis of current PV production and strategy for future silicon PV modules. (28th European Photovoltaic Conference and Exhibition, Paris, 2013).
79. Wang, J. *et al.* Large Chinese land carbon sink estimated from atmospheric carbon dioxide data. *Nature* **586**, 720–723 (2020).
80. Wene, C. *Stimulating Learning Investments through the "250 MW Wind"-Programme*. Available at: https://www.researchgate.net/publication/239868026 (1998).
81. Wesseh, Jr. P. K. & Lin, B. A real options valuation of Chinese wind energy technologies for power generation: do benefits from the feed-in tariffs outweigh costs?. *J. Clean. Prod.* **112**, 1591–1599 (2016).
82. Wild, M., Folini, D., Henschel, F., Fischer, N. & Muller, B. Projections of long-term changes in solar radiation based on CMIP5 climate models and their influence on energy yields of photovoltaic systems. *Sol. Energy* **116**, 12–24 (2015).
83. Williams, E., Hittinger, E., Carvalho, R. & Williams, R. Wind power costs expected to decrease due to technological progress. *Energy Policy* **106**, 427–435 (2017).
84. Xiang, C., Chen, F., Wen, F. & Song, F. Can China's offshore wind power achieve grid parity in time? *Int. J. Green Energy* **18**, 1219–1228 (2021).
85. Xiong, P. & Singh, C. Optimal planning of storage in power systems integrated with wind power generation. *IEEE T. Sustain. Energy* **7**, 232–240 (2015).
86. Xu, M., Xie, P. & Xie, B. Study of China's optimal solar photovoltaic power development path to 2050. *Resources Polic.* **65**, 101541 (2020).
87. Xu, X. China GDP spatial distribution kilometer grid data set. Available at: http://www.resdc.cn/DOI/ (2017).
88. Yan, J., Yang, Y., Campana, P. E. & He, J. City-level analysis of subsidy-free solar photovoltaic electricity price, profits and grid parity in China. *Nat. Energy* **4**, 709–717

(2019).

89. Yitzhaki, S. Relative Deprivation and the Gini Coefficient. *Q. J. Econ.* **93**, 321–324 (1979).

90. Yte1. Fuel Oil (180CST) price trend chart in 2022. Available at: http://www.yte1.com/datas/ranliaoyou-pri?end=2022 (2022).

91. Zhang, T., Emanuel, A. E. & Orr, J. A. Distribution feeder upgrade deferral through use of energy storage systems. *IEEE PESGM*, 16982692, 1–5, DOI: 10.1109/PESGM.2016.7968249 (2016).
